# Infliximab in chronic non-infectious paediatric uveitis refractory to previous biologic therapy

**DOI:** 10.1038/s41433-023-02795-3

**Published:** 2023-10-17

**Authors:** E. O. Kreps, S. J. Epps, A. Consejo, A. D. Dick, C. M. Guly, A. V. Ramanan

**Affiliations:** 1 Department of Ophthalmology, Ghent University Hospital, Ghent, Belgium; 2 Bristol Eye Hospital, University Hospitals Bristol and Weston, Lower Maudlin Street, Bristol, BS1 2LX United Kingdom; 3 Department of Applied Physics, University of Zaragoza, Zaragoza, Spain; 4 University College London, Institute of Ophthalmology, London, United Kingdom; 5 Academic Unit of Ophthalmology, Translational Health Sciences, University of Bristol, Bristol, UK; 6 NIHR Biomedical Research Centre of Ophthalmology, Moorfields Eye Hospital, London, UK; 7 Paediatric Rheumatology, Bristol Royal Hospital for Children and Translational Health Sciences, University of Bristol, Upper Maudlin Street, Bristol, BS2 8BJ United Kingdom

**Keywords:** Paediatrics, Uveal diseases

## Abstract

**Objectives:**

To examine the outcome of infliximab treatment in patients with non-infectious paediatric uveitis who have previously failed biologic treatment.

**Methods:**

A retrospective cohort study was performed at Bristol Eye Hospital, UK. Paediatric patients with chronic non-infectious uveitis who had been switched to infliximab due to inadequate uveitis control were identified. Two separate groups were evaluated: group 1 consisted of 20 children (36 eyes) who had been switched to infliximab following treatment failure with adalimumab (=in-class switching), while group 2 (5 patients; 9 eyes) included those who had been switched to infliximab from a non-TNF antagonist after failing several biologics (=across-class switching). The change in anterior chamber (AC) activity between baseline and 6- and 24-months follow-up was the primary outcome measure.

**Results:**

A statistically significant reduction in AC activity was found between baseline and 6-months follow-up (RE: *p* = 0.002; LE: *p* < 0.001) and between baseline and 24-months follow-up (RE: *p* = 0.016; LE: *p* = 0.011) in group 1. No statistically significant difference was found for either eye in the number of steroid eye drops needed between time points or the difference in visual acuity in time. In group 2, analysis of change of AC activity, number of steroid eye drops and visual acuity failed to reach statistical significance. Treatment failure occurred in four patients (20% of group 1) and adverse events developed in six patients including three patients with acute infusion reactions.

**Conclusions:**

This study supports the efficacy and safety of infliximab in adalimumab-refractory patients with paediatric non-infectious uveitis.

## Introduction

Persistent ocular inflammation in chronic, non-infectious paediatric uveitis confers a substantial risk of visual morbidity [1]. Methotrexate (MTX), a folate analogue inhibiting the enzyme dihydrofolate reductase, is usually used as an initial corticosteroid-sparing agent in childhood uveitis and is administered once a week orally or subcutaneously [1, 2]. It has been shown to be insufficiently effective in roughly one third of paediatric uveitis patients and up to 47% of children with juvenile idiopathic arthritis (JIA)-associated uveitis [2, 3]. In those with insufficient disease control, the common and licenced approach is to add adalimumab, a fully humanised monoclonal anti-TNFα antibody given subcutaneously once every two weeks. The SYCAMORE trial was a randomised controlled trial which showed the benefit of MTX combined with adalimumab for JIA-associated uveitis as compared with methotrexate alone [4]. Despite the clinical benefit, treatment failure still occurred in 27% of the group treated with MTX and adalimumab [4]. There is a scarcity of data to guide clinicians in managing this subset of children refractory to adalimumab combined with MTX. Consensus-based recommendations advise in class switching if uveitis is refractory to a first anti-TNFα although evidence to support this approach is limited [5]. Contrary to adalimumab, infliximab is an intravenously administered, chimeric human-mouse monoclonal IgG1 targeting TNFα and is not licensed in the UK for the treatment of uveitis. Previous case series have reported on the outcome of infliximab in paediatric uveitis with variable results [6]. This study aimed to assess whether paediatric patients with chronic non-infectious uveitis that have been refractory to a previous biologic therapy have a significant improvement in uveitis activity after switching to infliximab.

## Methods

A retrospective chart review was conducted in the paediatric uveitis service at Bristol Eye Hospital, United Kingdom. Paediatric patients who had been switched to infliximab treatment due to inadequate control of their uveitis on another biologic between January 1, 2011, and December 31, 2021 were identified. Patients with onset of chronic non-infectious uveitis before the age of 16 years, refractory to topical and/or systemic steroid treatment, at least 1 conventional systemic immunomodulatory treatment (IMT) (MTX, mycophenolate mofetil (MMF) and/or tacrolimus), and biologic agent, were eligible for inclusion. Refractory uveitis was defined as >1 + cells on >2 steroid drops per day for >3 months on current treatment. Patients with less than 6 months of follow-up after initiation of infliximab, or in which the switch to infliximab was to control systemic disease alone or with insufficient available data were excluded.

Two groups were assessed separately: group 1 included children who had failed adalimumab (as the sole prior biologic) and were subsequently switched to infliximab (=in-class switching) and group 2 contained those who had insufficient response to both adalimumab and other biologics and were switched to infliximab from a non-TNF antagonist (=across-class switching). Infliximab treatment was typically administered at a dose of 6 mg/kg with a dose at baseline, week 2, week 6 and then every 4–8 weeks depending on the clinical response. A standardised proforma was used to extract demographic and clinical information from ophthalmology and rheumatology medical records at baseline (i.e. at the time of the decision to switch to infliximab from another biologic) and 3, 6, 12 and 24 months following the switch to infliximab. Data collected at baseline visit included date of birth, sex, uveitis characteristics (onset date, diagnosis date, laterality, location), ocular complications and ocular surgeries. The anatomical location and grading of anterior chamber (AC) inflammation were in accordance with the Standardization of Uveitis Nomenclature (SUN) Classification Scale [7]. Adverse events and safety outcomes were also recorded and analysed. Controlled/quiescent uveitis was defined as an AC activity <1 + cells on ≤2 steroid eye drops per day. This maximum acceptable dose of topical steroids is based on research indicating an increased risk of IOP elevation [8] and cataract development [9] on 3 drops of prednisolone or more and aligns with the 2019 American College of Rheumatology (ACR) guidelines [10].

The primary outcome measure was the change in SUN grade of AC inflammation between baseline (the clinical examination prior to starting infliximab) and 6- and 24-months follow-up. This outcome measure was deemed appropriate for both anterior uveitis and the subset of intermediate and panuveitis as a flare of AC activity was the reason for switching to IFX in all patients included. Secondary outcome measures included the change in number of steroid eye drops (prednisolone acetate 1%), change in best-corrected visual acuity, the occurrence of cystoid macular oedema and the safety of infliximab treatment. Acute infusion reactions were defined as any adverse event occurring during the infusion or within 1 hour of termination [11].

All extracted data was tabulated in Microsoft Excel (version 16.16.10, Microsoft Corp, Redmond, WA, USA) and analysed using SPSS software for Windows version 25.0 (SPSS Inc., Chicago, Illinois, United States). Spearman’s rank-order correlation test was applied to anterior chamber activity (ordinal data from the SUN grading) to investigate the degree of correlation between right and left eyes [12]. The non-parametric one-way repeated measures Friedman test was conducted to test for time differences (baseline, 6-months follow up, and 24-months follow-up) for anterior chamber activity and the number of steroid eye drops needed in groups 1 and 2 separately. A post-hoc test for multiple comparisons was applied (Wilcoxon signed-rank test) with Bonferroni correction; for a 0.05 significance level, the alpha error per comparison was set to 0.017 (0.05/3). One-way repeated measures ANOVA test was performed to ascertain whether visual acuity changed over time (baseline, 6-months follow up, and 24-months follow-up). Shapiro-Wilk test, Mauchly’s test of sphericity, and Levene’s test indicated that the assumptions of normality, sphericity and homogeneity of variances, respectively, had not been violated. The level of significance was set to 0.05 unless stated otherwise.

## Results

Of the 28 eligible patients identified, 25 (89.3%) patients (45 eyes) were included: 20 patients (36 eyes) in group 1 and five (9 eyes) in group 2. In two patients, the switch to infliximab had been managed locally and insufficient data could be retrieved, and another patient had not reached 6 months of follow-up since the first infliximab dose. As part of a preliminary analysis, moderate to high correlation was found between right and left eyes in some instances under investigation (Group 1: baseline: *ρ* = 0.40, *p* = 0.12; 6-months follow-up: *ρ* = 0.54, *p* = 0.045, and 24-months follow-up: *ρ* = 0.68, *p* = 0.06 ; Group 2: baseline: *ρ* = −0.24, *p* = 0.77; 6-months follow-up: *ρ* = 1.0, *p* = n/a, and 24-months follow-up: *ρ* = −0.24, *p* = 0.76; Spearman’s rank-order correlation test). Consequently, to reduce the risk of Type 1 errors, the right and left eyes were assessed separately. A summary of the patients’ characteristics is provided in Table 1. All patients had received conventional IMT prior to and in conjunction with their previous biologic. Fourteen (56%) patients had been switched to MMF during follow-up due to intolerance to MTX. Every patient used at least one IMT in conjunction with infliximab; in five patients (20%), a combination of MTX and MMF was prescribed. Two patients, both with Blau syndrome, received frequent IV methylprednisolone treatment in conjunction with their infliximab infusions because of difficult-to-control uveitis. One of these patients with Blau syndrome was also the only patient on low-dose systemic prednisolone treatment while on infliximab treatment.

**Table 1:** Demographic and clinical data of included patients (total group) and both subgroups (JIA: juvenile idiopathic arthritis).

	Group (Total)	Group 1	Group 2
Patients (number of eyes)	25 (45 eyes)	20 (36 eyes)	5 (9 eyes)
Age at uveitis diagnosis—median (range)	6 years (1–15)	7 years (1–15)	4 years (2–10)
Sex			
Male	11 (44%)	10 (50%)	1 (20%)
Female	14 (56%)	10 (50%)	4 (80%)
Laterality			
Bilateral	20 (80%)	16 (80%)	4 (80%)
Unilateral	5 (20%)	4 (20%)	1 (20%)
Uveitis—anatomic location			
Anterior	21 (84%)	16 (80%)	5 (100%)
Intermediate	1 (4%)	1 (5%)	0
Posterior	0	0	0
Panuveitis	3 (12%)	3 (15%)	0
Systemic association			
JIA	16 (64%)	11 (55%)	5 (100%)
Blau syndrome	2 (8%)	2 (10%)	0
None	7 (28%)	7 (35%)	0
IMT prior to infliximab			
Methotrexate	24 (96%)	19 (95%)	5 (100%)
Mycophenolate Mofetil	14 (56%)	11 (55%)	3 (60%)
Tacrolimus	3 (12%)	2 (10%)	1 (20%)
Previous biologic treatment			
Adalimumab	25 (100%)	20 (100%)	5 (100%)
Tocilizumab	4 (16%)	0	4 (80%)
Etanercept	1 (4%)	0	1 (20%)
Abatacept	1 (4%)	0	1 (20%)
Baricitinib	1 (4%)	0	1 (20%)
Duration of biologic treatment pre-infliximab—median (range)	26 months (7-83)	26 months (7-83)	28 months (20–42)
Age at switch to infliximab—median (range)	13 years (2.5–17)	14 years (2.5–17)	10 years (7–12)
Duration of infliximab treatment—median (range)	36 months (4–114)	25 months (4–114)	68 months (28–98)
Co-medication			
Methotrexate	16 (64%)	13 (65%)	3 (60%)
Mycophenolate Mofetil	14 (56%)	11 (55%)	3 (60%)
Frequency of infliximab^a^			
Every 4 weeks	13 (52%)	10 (50%)	3 (60%)
Every 6 weeks	8 (32%)	7 (35%)	1 (20%)
Every 8 weeks	4 (16%)	3 (15%)	1 (20%)

The median of intervals was registered for each patient.

^a^Frequency of infusions in the first 24 months.

An overview of the treatment response to infliximab is provided in Tables 2 (Group 1) and 3 (Group 2).

**Table 2:** Clinical data at baseline visit and follow-up of infliximab treatment of group 1 (in-class switch). Nine patients (17 eyes) did not complete the 24-month follow-up period: four patients experienced treatment failure, one encountered an infusion reaction, and four patients were still undergoing infliximab treatment beyond the 6-month mark but had not yet reached the 24-month time point. (AC anterior chamber, VA visual acuity, RE right eyes, LE left eyes).

					*P* values	
Group 1		Baseline	6 months	24 months	RE	LE
AC	1+ or more	83.3% (30/36 eyes)	15.6% (5/32 eyes)	31.6% (6/19 eyes)	0.004^a^	<0.001^a^
	3+ or 4+	33.3% (12/36 eyes)	0% (0/32 eyes)	0% (0/19 eyes)		
Steroid drops	1 or less	16.7% (6/36 eyes)	56.3% (18/32 eyes)	73.7% (14/19 eyes)	0.22^a^	0.053^a^
	>1	83.3% (30/36 eyes)	43.8% (14/32 eyes)	26.3% (5/19 eyes)		
VA	<6/12	22.2% (8/36 eyes)	12.5% (4/32 eyes)	21.1% (4/19 eyes)	0.23^b^	0.64^b^
	<6/60	8.3% (3/36 eyes)	0% (0/32 eyes)	0% (0/19 eyes)		

Please see text for more detailed statistical analysis.

^a^one-way repeated measures Friedman test.

^b^one-way repeated measures ANOVA test.

**Table 3:** Clinical data at baseline and follow-up of infliximab treatment of group 2 (between-class switch) (AC anterior chamber, VA visual acuity, RE right eyes, LE left eyes).

					*P* values	
Group 2		Baseline	6 months	24 months	RE	LE
AC	1+ or more	88.9% (8/9 eyes)	22.2% (2/9 eyes)	11.1% (1/9 eyes)	0.012^a^	0.038^a^
	3+ or 4+	44.4% (4/9 eyes)	0% (0/9 eyes)	0% (0/9 eyes)		
Steroid drops	1 or less	0% (0/9 eyes)	66.7% (6/9 eyes)	66.7% (6/9 eyes)	0.092^a^	0.15^a^
	>1	100% (9/9 eyes)	33.3% (3/9 eyes)	33.3% (3/9 eyes)		
VA	<6/12	11.1% (1/9 eyes)	11.1% (1/9 eyes)	0% (0/9 eyes)	0.70^b^	0.30^b^
	<6/60	0% (0/9 eyes)	0% (0/9 eyes)	0% (0/9 eyes)		

Please see text for more detailed statistical analysis.

^a^one-way repeated measures Friedman test.

^b^one-way repeated measures ANOVA test.

### Group 1 (In-class switching)

A statistically significant difference in AC activity between time points (baseline, 6-months follow up and 24-months follow up) was found for both eyes (RE: *χ*^2^ = 11.08, *p* = 0.004; LE: *χ*^2^ = 15.93, *p* < 0.001). The corresponding post-hoc test revealed that AC activity was statistically significant different between baseline and 6-months follow-up (RE: *p* = 0.002; LE: *p* < 0.001) and between baseline and 24-months follow-up (RE: *p* = 0.016; LE: *p* = 0.011). There was no statistically significant difference in anterior chamber activity between 6-months and 24-months follow-up (RE: *p* = 0.26; LE: *p* = 0.18). No statistically significant difference in the number of steroid eye drops needed between time points (baseline, 6-months follow-up, and 24-months follow-up) was found for either eye in group 1 (RE: *χ*^2^ = 3.00, *p* = 0.22; LE: *χ*^2^ = 5.87, *p* = 0.053). No statistically significant difference in visual acuity in time (baseline, 6-months follow up and 24-months follow-up) was found for either eye in group 1 (RE: F(2,18) = 1.52, *p* = 0.23, *ƞ*_p_^2^ = 0.42; LE: F(2,18) = 0.46, *p* = 0.64, *ƞ*_p_^2^ = 0.36).

### Group 2 (across-class switching)

Differences in AC activity between time points (RE: *χ*^2^ = 8.82, *p* = 0.012; LE: *χ*^2^ = 6.53, *p* = 0.038) were statistically significant, however, when taking Bonferroni correction into account in post-hoc testing, most differences lost their statistical significance: AC activity between baseline and 6-months follow-up (RE: *p* = 0.041; LE: *p* = 0.066), between baseline and 24-months follow-up (RE: *p* = 0.043; LE: *p* = 0.063), and between 6-months and 24-months follow-up (RE: *p* = 1.00; LE: *p* = 0.56). The difference in number of steroid eye drops needed between time points was not statistically significant (RE: *χ*^2^ = 4.77, *p* = 0.092; LE: *χ*^2^ = 3.80, *p* = 0.15), and neither was the difference in visual acuity for either eye (RE: F(2,8) = 0.40, *p* = 0.70, *ƞ*_p_^2^ = 0.37; LE: F(2,8) = 1.38, *p* = 0.30, *ƞ*_p_^2^ = 0.27).

Ocular complications were present at baseline (pre-infliximab) in most patients (Table 4). In 7 eyes in which cataracts had developed prior to infliximab treatment, quiescence of uveitis on infliximab (<1 + cells for >3 months) allowed for surgical intervention with a short course of oral corticosteroids of 4-6 weeks as perioperative cover. No patients developed glaucoma or ocular hypertension while on infliximab. Glaucoma surgery was performed in two eyes of two patients with pre-existing glaucoma while on infliximab. During infliximab treatment, two patients (1 intermediate uveitis and 1 Blau syndrome) developed a unilateral rhegmatogenous retinal detachment which required both scleral buckling and pars plana vitrectomy with oil tamponade.

**Table 4:** Ocular complications and surgical procedures pre and during IFX treatment expressed as incidence rate (IR) per year of follow-up at the ocular level. (IFX Infliximab, PPV pars plana vitrectomy).

	Group 1				Group 2			
	Pre-IFX		During IFX^a^		Pre-IFX		During IFX^a^	
COMPLICATIONS	N eyes (%)	IR	N eyes (%)	IR	N eyes (%)	IR	N eyes (%)	IR
Band keratopathy	12 (33.3%)	0.05	0 (0%)	0	1 (11.1%)	0.02	0 (0%)	0
Posterior synechiae	30 (83.3%)	0.13	0 (0%)	0	4 (44.4%)	0.09	0 (0%)	0
Cataract	17 (47.2%)	0.07	0 (0%)	0	3 (33.3%)	0.06	0 (0%)	0
Ocular hypertension	13 (36.1%)	0.06	0 (0%)	0	4 (44.4%)	0.09	0 (0%)	0
Glaucoma	7 (19.4%)	0.03	0 (0%)	0	4 (44.4%)	0.09	0 (0%)	0
Disc swelling	5 (13.9%)	0.02	1 (2.8%)	0.01	2 (22.2%)	0.04	0 (0%)	0
Macular oedema	5 (13.9%)	0.02	2 (5.6%)	0.02	2 (22.2%)	0.04	1 (11.1%)	0.02
Retinal detachment	0 (0%)	0	2 (5.6%)	0.02	0 (0%)	0	0 (0%)	0
SURGERY								
Cataract surgery	6 (16.7%)	0.03	4 (11.1%)	0.04	0	0	3 (33.3%)	0.06
Trabeculectomy	4 (11.1%)	0.02	0	0	2 (22.2%)	0.04	2 (22.2%)	0.04
Tube surgery	1 (2.8%)	0.004	0	0	0	0	0	0
PPV with buckle	0 (0%)	0	2 (5.6%)	0.02	0	0	0	0

^a^new onset or recurrence whilst on infliximab treatment.

Treatment failure that resulted in cessation of IFX occurred in four patients (all in group 1 (20%); 3 JIA-associated uveitis and 1 Blau syndrome), after 4 months (*n* = 2), 18 months (*n* = 1) and 42 months (*n* = 1) respectively. Adverse events developed in six patients of group 1 (30%) and no patients of group 2. Three patients had acute infusion reactions: breathlessness and wheezing (n = 2; after 9 and 15 months of treatment) and mild chest pain during infusion (*n* = 1). In the former two patients, infliximab treatment was stopped. In the latter, work-up including anti-infliximab antibody analysis was negative and no new issues arose upon continuation of treatment. Two patients had frequent infections, often requiring antibiotics (urinary tract infections in one, and upper respiratory tract infections in the second patient) and one patient had a transient elevation of liver transaminases which settled upon withholding methotrexate for 3 weeks.

## Discussion

In this study, infliximab was effective at achieving disease control in paediatric patients with non-infectious uveitis who were refractory to prior biologic treatment including adalimumab. Statistical significance was reached for the primary endpoint (AC activity) at the 6- and 24-month timepoints for the group of 20 patients switched from adalimumab, but not for the small group of five patients. Due to the low number of patients in this second group, however, this result should be interpreted with caution. The secondary treatment endpoints (steroid drops and visual acuity) also showed improvement but did not attain statistical significance, neither did analysis of the small group of five patients who switched from non-TNFα biologic. At a patient level, bilateral uveitic control (AC activity <1 + cells on <2 steroid eye drops per day for all eyes involved) was achieved in 72.2% after 6 months of infliximab treatment following adalimumab failure, which was maintained at the 24-month time point. A previous study also reported excellent treatment response to infliximab, with steroid-remission on infliximab treatment (5–10 mg/kg/dose 4–6 weekly) in 69% of 13 adalimumab-refractory patients with chronic non-infectious uveitis [13]. These data support the efficacy of infliximab – despite targeting the same TNFα cytokine—in adalimumab-refractory patients. In biologic treatment-naïve patients with childhood uveitis, a similar average response rate of infliximab of 72% was calculated by Simonini et al. in their meta-analysis [6]. As compared to adalimumab, infliximab has wider dosing customisation in both dose and frequency of administration, which may contribute to the efficacy in adalimumab-refractory patients. In the overall cohort of 25 patients in this study, 52% required 4-weekly administration of infliximab at a dose of 6 mg/kg/dose, with an additional eight patients (32%) receiving 6-weekly infusions. Studies on the outcome of infliximab in paediatric uveitis in which less favourable results were reported, have frequently utilised doses of 3–5 mg/kg/dose every 6–8 weeks [14–17]. Clinicians need to take this issue into account when assessing treatment response to infliximab, in differentiating insufficient dosing and/or premature extension of infliximab from true infliximab failure.

No patients developed new-onset cataract and glaucoma whilst on infliximab treatment and quiescence of uveitis allowed for surgical intervention of pre-existent cataract. Peripheral vitreous traction in intermediate or panuveitis can seldomly result in rhegmatogenous retinal detachment [18, 19]. Both patients who developed a unilateral retinal detachment in this study initially had scleral buckle surgery but required pars plana vitrectomy with oil tamponade 1-2 weeks following the first surgery. Large series of paediatric retinal detachments similarly indicate that most patients require scleral buckling and pars plana vitrectomy, with oil tamponade being the most frequently used in the paediatric population [20, 21]. Three patients (12%) experienced acute infusion reactions (with full recovery), which led to cessation of infliximab treatment in two patients. This rate aligns with other series in which a dose of 6 mg/kg/dose 4–8 weekly and co-medication with conventional immunomodulatory treatment (mainly MTX) was used [22, 23]. Anti-infliximab antibodies are associated with both secondary loss of clinical response and a significant increased risk of drug reactions [23, 24]. Both higher and more frequent dosing—such as appears to be indicated in uveitis—as well as routine co-medication with conventional IMT have been shown to be protective against antibody formation [11, 23, 24].

Several limitations of this study need to be considered. The design as a retrospective, single-centre uncontrolled study with heterogeneity of disease and duration of follow-up renders it at risk of bias and confounding. During follow-up, 56% of patients were also switched to MMF due to MTX intolerance, which may have impacted disease control as well. Anti-infliximab antibodies and drug levels were not routinely measured. Therapeutic options not requiring IV administration, including weekly adalimumab and/or subcutaneous infliximab, were not assessed in this study. Treatment adherence, which has previously been shown to impact the odds of having active disease on infliximab, could not be assessed due to the retrospective nature of the study [25].

The data presented in this study indicates that, in cases where a child’s uveitis remains active despite systemic IMT in combination with a biologic agent, switching to infliximab is effective and well-tolerated. A larger, multicentre, prospective study, with measurement of trough infliximab levels and anti-infliximab antibodies, is warranted to address this question more robustly and help determine patient-specific dosing of infliximab for the indication of uveitis.

## Summary

### What was known before

- Adalimumab is the only biologic licenced for use in non-infectious childhood uveitis in the UK. Little data is available on the efficacy of infliximab in adalimumab-refractory patients.

### What this study adds

- Infliximab is an effective and well-tolerated treatment for adalimumab-refractory paediatric uveitis.

## Data Availability

All data generated or analysed during this study are included in this published article.
